# Experimental–theoretical analysis of cooling and freezing of a droplet in contact with a cold substrate: influence of substrate wettability

**DOI:** 10.1098/rsta.2024.0363

**Published:** 2025-07-17

**Authors:** Prasenjit Kabi, Simrandeep Bahal, Manish K. Tiwari, Emerson Barbosa dos Anjos, Carolina Palma Naveira-Cotta, Renato Machado Cotta

**Affiliations:** ^1^ Nanoengineered Systems Laboratory, Mechanical Engineering, University College London, London WC1E 7JE, UK; ^2^ UCL Hawkes Institute, University College London, London W1W 7TS, UK; ^3^ Laboratory of Nano and Microfluidics and Microsystems, LabMEMS, Mechanical Engineering Dept., POLI and COPPE, UFRJ, Federal University of Rio de Janeiro, Rio de Janeiro, Brazil; ^4^ IPqM-CTMRJ, General Directorate of Nuclear and Technological Development, DGDNTM, Brazilian Navy, Rio de Janeiro, Brazil

**Keywords:** freezing, icing, reduced model, infrared thermography, improved lumping, wettability

## Abstract

The physics and modelling of cooling and freezing of droplets in contact with a colder substrate are of interest in various engineering applications. This work provides experimental results of this process employing infrared thermography for temperature measurements at the droplet’s surface. Also, a high-speed camera is employed to observe the recalescence period and measure the freezing front movement and the droplet shape change. Three substrates are prepared with distinct wettability ranges, i.e. one hydrophilic and two hydrophobic surfaces. From the experimental observation of a solidification front parallel to the substrate plane, a mixed lumped-differential model of the heat transfer process based on the Coupled Integral Equations Approach is proposed, reformulating the two-dimensional partial differential formulation in cylindrical coordinates into a one-dimensional transient energy equation for the droplet external surface temperature. Direct comparisons of the experimental and theoretical results for the supercooling period show excellent agreement for the droplet surface temperatures at different heights and for different values of the substrate–droplet contact angle. It is also shown that the classical partial lumped system analysis does not provide adequate predictions in the present problem. Finally, the dynamics of the recalescence and freezing stages are experimentally evaluated and physically interpreted.

This article is part of the theme issue ‘Heat and mass transfer in frost and ice’.

## 1. Introduction

Water exists as a liquid far below its thermodynamic freezing temperature (super cooled) but readily freezes when in contact with a sufficiently cold solid surface. This is due to the lowered barrier of heterogeneous nucleation compared with homogeneous nucleation [1]. While suppression and removal of frost in closed systems, such as in HVAC applications, has been achieved to some extent [2], atmospheric ice accretion remains a matter of grave concern for aviation [3–6], maritime transport [7–9], wind turbine [10–12], power transmission [13–15] and road transport [16,17] sectors. De-icing by heating and/or vibrating are effective [18] but energy and time consuming [19–21]. At the same time, the use of freezing point depressants are effective passive strategies albeit with an environmental penalty [22,23]. Where applicable, the development of liquid repellent and/or photo thermal [20] coatings are promising but yet to be commercialized. Ice accretes through impact of super cooled droplets or solidification of condensed vapour. A better understanding of atmospheric icing, through observing a super cooled droplet freezing process, is quite desirable towards designing effective icephobic strategies.

**Figure 1.**
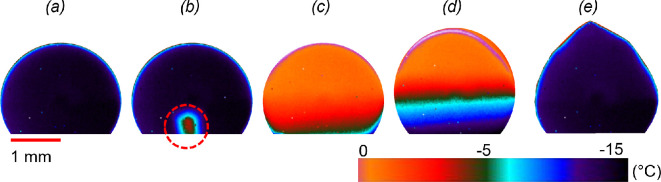
Sequence of side view images showing the spatial and temporal evolution of the temperature field of a 20 µl water droplet. (*a*) A super cooled droplet, (*b*) onset of recalescence (dashed circle) approximately 5 ms later, (*c*) completion of recalescence approximately 40 ms later, (*d*) approximately 7 s post-recalescence, the second stage of freezing is shown by the moving ice front, (*e*) end of freezing (14 s post-recalescence) of the water droplet characterized by a sharp tip at the apex.

Solidification of a liquid sessile droplet is a complex interplay of several physical factors but may be described in four stages [24] as shown in figure 1: (*a*) *supercooling—*the sessile droplet is cooled below its thermodynamic freezing temperature (*T* < *T*_e_) as shown in figure 1, depending on the substrate’s surface roughness, wettability [25] and ambient conditions [26–28]; (*b*) *nucleation—*a stable ice embryo forms, either at the liquid–substrate interface or the liquid–air interface [29] by overcoming the Gibbs’ energy barrier to heterogeneous nucleation [1]. The temperature at which nucleation occurs is an important characteristic of icephobic surface; (*c*) *recalescence—*proliferation of ice nuclei within the droplet accompanied by a rapid rise in its bulk temperature to *T*_e_. The droplet is now a frozen mixture of ice and water where the ice concentration is estimated by balancing the release of latent heat to the sensible heat required to raise the droplet’s temperature. The entire process is assumed near adiabatic since the duration of recalescence ($∼\text{O}\text{(}{\text{10}}^{\text{-2}}\text{)}\text{s)}$ is considerably shorter than the thermal diffusion timescale $\approx \frac{{\text{R}}_{\text{drop}}^{\text{2}}}{\rho_{\text{water}}{\text{C}}_{\text{p}}}{\text{k}}_{\text{water}}∼O\left(10\right)s$. Here *R*_drop_ is the nominal size of a liquid droplet (~O(1) mm), thermal conductivity of water (*k*_water_) is considered as 0.607 W/m.K, density as 997 kg m^−3^ and specific heat capacity (*C*_p_) as 4.2 J/g.K; (*d*) *solidification—*starting at the droplet’s base and moving in the direction imposed by the temperature gradient [29–31]; (*e*) finally, solidification culminates (in case of water) into a conical tip at the apex [32,33]. Solidification is limited by the rate of heat transfer to the ambient [34], after which the droplet cools down to the substrate temperature.

Icephobic surfaces usually focus on delaying the onset of nucleation (freezing delay). Freezing delays up to 25 h [35,36] have been reported. However, atmospheric conditions can trigger nucleation at the liquid–air interface [29] and render icephobic surfaces useless. Solidification or the freezing time should be equally important; a frozen ice droplet is harder to remove, likely to anchor other droplets impinging on the surface and accelerate frost formation [37]. The freezing time can be in principle estimated from the one-dimensional Stefan’s model of solidification [38]. Attempts of theoretically analysing the ice formation process include classical numerical techniques such as the enthalpy method [24,39], enthalpy-porosity technique [34], front tracking technique [40] and heat capacity method [31]. A fairly complete review was provided by Akhtar *et al.* [41], discussing the various mathematical approaches undertaken to model all stages of droplet freezing. Two less known hybrid numerical–analytical approaches in the droplet freezing field have been recently adopted to simulate the four stages of a suspended droplet freezing process [42–44]. One of them is the Generalized Integral Transform Technique or GITT [45–52], which is a hybrid numerical–analytical approach based on the classical integral transform method for linear partial differential equations, but generalized to handle nonlinear formulations, including moving boundary problems. The GITT approach is demonstrated [42] for one-dimensional transient droplet freezing, adopting a nonlinear eigenvalue problem proposal [51] with markedly improved convergence rates. Accurate benchmark results were then generated and used to co-validate experimental results for a suspended droplet from Hindmarsh *et al*. [53]. The same hybrid approach was employed in the analysis of a two-dimensional heat conduction formulation for a droplet in contact with a cold superhydrophobic substrate [43]. The second approach considered for simulating freezing of a suspended droplet [44] is in fact a problem reformulation strategy, known as the Coupled Integral Equations Approach (CIEA) [52,54–58], which provides improved lumped-differential formulations by appropriate averaging the full partial differential problem in one or more spatial coordinates, and using information from the boundary conditions in the Hermite formulae for the approximated averaged quantities, such as temperatures and heat fluxes. Such improved lumped formulations were then tested on the same suspended droplet freezing problem [43,44] and compared against the same set of experimental results [53]. Experimental co-validation with Stefan-type models is based on optical imaging of the moving ice front [28,31] and measuring the droplet temperature through both intrusive [24,26,59] and non-intrusive techniques [24,34,60]. Carefully calibrated infra-red measurements [34] are usually better for being non-intrusive, thus avoiding nucleation and heat conduction from within the droplet and providing better spatio-temporal resolution.

In the present work, first the cooling and freezing of a supercooled droplet in contact with a cold substrate is analysed experimentally. Non-intrusive infrared thermography is employed throughout the process to accurately measure the droplet surface temperatures, while a high-speed camera is used to determine the freezing front evolution. Different substrate surfaces have been prepared to provide a range of wettability behaviours, spanning from hydrophilic to superhydrophobic characteristics. Based on the observed almost vertical advance of the freezing front, a lumped-differential model has been proposed, using the CIEA [52,54–58]. Considering the droplet as a deformed cylinder, with a variable external radius as a function of the vertical coordinate, a lumping process is proposed in the radial coordinate only, thus transforming the axisymmetric two-dimensional partial differential formulation for the entire droplet, into a one-dimensional one for the droplet temperatures at the external surface. Then, the theoretical predictions are critically compared with the experimentally determined surface temperatures along the droplet cooling phase, at different vertical positions and for the three different substrate preparations. Finally, the experimental behaviour of the entire freezing process, including recalescence, is illustrated and compared for the three types of substrates. Please see table 1 for nomenclature.

**Table 1.** Nomenclature.

B(θ)	auxiliary parameter of the nonlinear boundary condition
Bi_c_	Biot number for contact heat transfer between droplet and substrate surface
Bi	Biot number for convective heat transfer between droplet and air
Bim	dimensionless group for mass transfer
Bir	dimensionless group for radiative heat transfer
*c*	specific heat (J kg^−1^ K^−1^)
*D*	droplet diameter (m)
*D_a_*	diffusivity (m^2^ s^−1^)
g	gravity acceleration (m s^−2^)
*h*	heat transfer coefficient (W m^−2^ K^−1^)
*h_m_*	mass transfer coefficient (m s^−1^)
*k*	thermal conductivity (W m^−1^ K^−1^)
*L_1_*	liquid droplet height (mm)
*L_2_*	liquid droplet base diameter (mm)
*L_e_*	latent heat of evaporation (J kg^−1^)
*Nu*	Nusselt number
*n_z_*	direction cosines of the unit normal vector with respect to the *z* coordinate
*n_r_*	direction cosines of the unit normal vector with respect to the *r* coordinate
*Pr*	Prandtl number
*q*	heat flux (W m^−2^)
*R_d_*	initial spherical droplet radius (m)
*Re*	Reynolds number
*R_H_*	relative humidity
*r*	radial coordinate (m)
*r_w_*	droplet surface radius along axial variable (m)
*R*	dimensionless radial variable
*S(t*)	freezing front position (mm)
*Sc*	Schmidt number
*Sh*	Sherwood number
*t*	time variable (s)
*T(r,z,t*)	temperature distribution (K)
*T_n_*	nucleation temperature (K)
*T_0_*	initial temperature (K)
*T_b_*	substrate temperature (K)
*T_w_*	temperature distribution on the droplet surface (K)
*T_∞_*	ambient air temperature (K)
*Z*	dimensionless axial variable
*z*	axial variable
*Greek symbols*	
*α*	thermal diffusivity (m^2^ s^−1^)
$\beta_{\infty }$	volumetric expansion coefficient (1 K^−1^)
*ε*	emissivity
*θ(R,Z,τ*)	dimensionless temperature in cylindrical coordinates
$\bar{\theta }$(*Z,τ*)	average dimensionless temperature
$\tilde{\theta }(Z,\tau )$	auxiliary dependent variable
$\mu_{\infty }$	dynamic viscosity of air (N s m^−^²)
*ρ*	density (kg m^−3^)
*ρ_v,0_*	water vapour density at 273 K (kg m^−3^)
*ρ_v,l_*	water vapour density at liquid droplet surface (kg m^−3^)
*ρ_v,∞_*	water vapour density at surrounding air temperature (kg m^−3^)
*σ*	Stefan–Boltzmann constant (W m^−2^ K^−4^)
*τ*	dimensionless time variable
Λ	dimensionless parameter (related to the front position)
ν	kinematic viscosity (m^2^ s^−1^)
γ	ratio between the radius of the base and the height of the droplet
*Subscripts and superscripts*	
∞	air
av	average
b	base-substrate
c	related to droplet–substrate contact
*m*	related to convective mass transfer
*r*	related to radiative heat transfer
*ice*	solid phase
*l*	liquid phase
*w*	wall (substrate)
1 to 4	stage 1 (supercooling), 2 (recalescence), 3 (freezing), 4 (cooling)

## 2. Experimental methods

Aluminium sheets (grade 6061) 1.5 mm thick are cut into 2.5 cm × 2.5 cm coupons. They are degreased by sequentially washing in acetone, iso-propanol, ethanol and water baths. The degreased samples are kept aside to be used as hydrophilic substrates (denoted as HYL in the text). The rest is dipped in 3 M KOH solution to remove the native oxide layer before being anodized in 0.3 M oxalic acid solution. See Grizen *et al.* [61] for details. The anodized samples are then immersed in 1 wt% solution of octadecyltricholosilane in hexane for 1 h and subsequently heated at 120°C in a temperature-controlled oven for 2 h. The static contact angle on the HYL sample is approximately 80 ± 5°. For the silane-treated anodized sample (referred to in the text as HYB), the static contact angle is approximately 113 ± 5°. In order to decrease the area of substrate–droplet contact, some of the anodized samples are further immersed in 0.3 M phosphoric acid solution for 2 h with constant stirring. This leads to wider anodic pores and combined with the silane treatment gives a static contact angle of approximately 135 ± 5°. This substrate is referred to as SHB.

**Figure 2.**
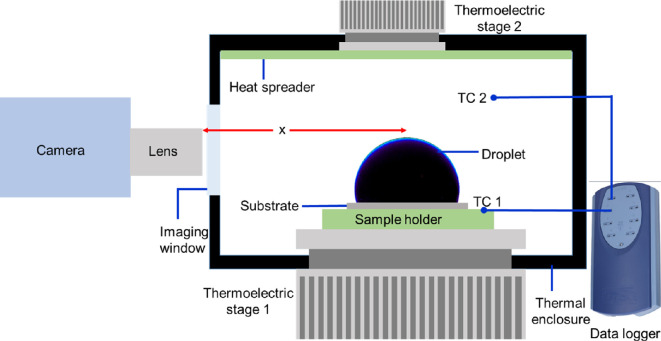
Schematic of the experimental set-up. The thermoelectric stage 1 is CP-065, thermoelectric stage 2 is a generic Peltier module. TC 1 and TC 2 are used to monitor substrate and air temperature, respectively. The camera used is either ×901sc (FLIR) with a MIC 1× lens for thermal imaging or v411 (Phantom) with a K2 Distamax lens. The distance between the lens and droplet is denoted as *x* and equals 30 mm for the IR imaging and 98 mm for optical imaging.

The substrate (HYL or HYB or SHB) is placed in a three-dimensional printed enclosure (40 mm × 40 mm × 40 mm), in the experimental schematic shown in figure 2. An air cooled thermo-electric cooler (CP-065, TE Technology) is used for all experiments. The topside of the enclosure is fitted with the thermoelectric module (with a heat spreader) to further cool the air inside the enclosure to −1.5 ± 1°C. Two thermocouples are used to measure the substrate temperature (positioned beside the actual substrate) and air temperature (position 10 mm from the substrate). Thermocouple readings are logged using a TC-08 (USB temperature logger, Picolog) at 1 Hz. Prior to the experiments, the thermocouples are calibrated in a re-circulating chiller bath at different temperatures in the range of interest.

Thermal infra-red (IR) images are acquired using the ×901sc camera (FLIR) fitted with a MIC 1× microscope lens (pixel resolution of 40 µm). The distance between the lens and the droplet is *x* = 30 mm. In order to avoid spurious background effects, the inside of the enclosure is covered with a black masking tape (Thorlabs). A circular window covered with IR transparent sheet of polypropylene is used to view the droplets. The acquisition frame rate is maintained at 1 frame per second (fps) till the substrate temperature is below −10°C and then continued at 400 fps till the end of freezing. Error due to emissivity and view factor was found to be between −1 and −2°C, which is within 10% of the temperature range measured in this current work. A constant emissivity of 1 was used for all IR image acquisitions.

To better visualize the icing front, separate freezing experiments (under the same conditions) are imaged using a high-speed camera (Phantom v411) fitted with a long-distance microscope (K2 Distamax + CF2 lens, Infinity Photo-Optical, USA) with spatial resolution of 7 µm. The distance between the lens and the droplet is *x* = 98 mm. Two light sources are used, one opposite to the camera to outline the droplet profile and the other kept perpendicular to illuminate the droplet interior. Images are acquired at 100 fps.

The following procedure is followed for all experiments. The droplet is dispensed using a handheld pipette at room temperature and the enclosure is secured. The thermoelectric modules are switched on and thermal images are continuously acquired as described above. For optical images, the camera is triggered 2 s before recalescence (image-based trigger available on Phantom cameras).

## 3. Problem formulation and solution

**Figure 3.**
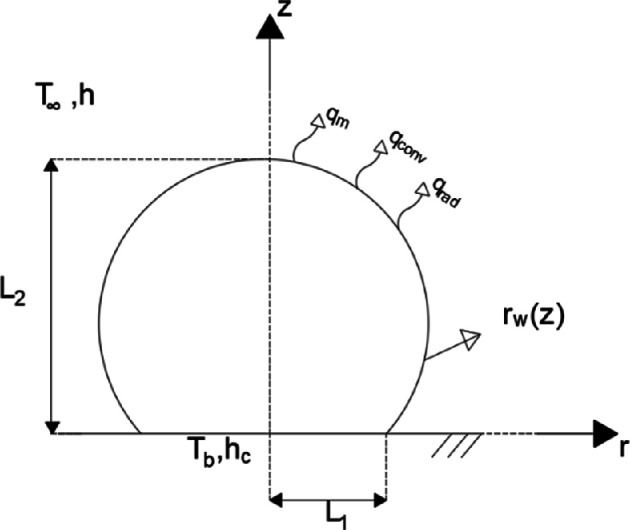
Configuration of a droplet supported on a cold surface. Mathematical model in cylindrical coordinates–spheroidal solids.

The supercooling stage (first stage) for the sessile droplet in contact with a cold substrate is discussed here. The transient two-dimensional heat conduction equation is used, with the mathematical formulation written in cylindrical coordinates (for a deformed cylinder with external radius variable with the axial coordinate; see figure 3) with proper initial and boundary conditions given by:


(3.1a) $$\;\frac{1}{\alpha }\frac{\partial T(r,z,t)}{\partial t}=\frac{1}{r}\frac{\partial }{\partial r}\left(r\frac{\partial T(r,z,t)}{\partial r}\right)+\frac{\partial^{2}T(r,z,t)}{\partial z^{2}}\;at\;[\begin{array}{c} 0<z<L_{2}\\ 0<r<r_{w}(z)\\ t>0 \end{array}$$

Initial condition:


(3.1b) $$T\left(r,z,0\right)=T_{0}$$

Boundary conditions:


(3.1c,d) $${\frac{\partial T(r,z,t)}{\partial z}|}_{z=L_{2}}=0,\;{-k\frac{\partial T(r,z,t)}{\partial z}|}_{z=0}+h_{c}T(0,z,t)=h_{c}T_{b}$$

(3.1e) $${\frac{\partial T(r,z,t)}{\partial r}|}_{r=0}=0$$

(3.1f) $$-k\left({n_{r}\frac{\partial T(r,z,t)}{\partial r}|}_{r=r_{w}(z)}+{n_{z}\frac{\partial T(r,z,t)}{\partial z}|}_{r=r_{w}(z)}\right)=h\left(T-T_{\infty }\right)+h_{m}L_{e}\left(\rho_{v,l}(T)-\rho_{v,\infty }\right)+\varepsilon \sigma \left(T^{4}-T_{\infty }^{4}\right).$$

where *L*_1_ is the liquid droplet base diameter, *L*_2_ is the liquid droplet height, α (m² s^−1^) is the thermal diffusivity of water, *T*(*r*,*z*,*t*) is the droplet temperature, *t* is the time, *r* is the radial coordinate, *z* is the axial coordinate, *T*_0_ is the initial droplet temperature, *k* (W m^−1^K^−1^) is the liquid thermal conductivity, *h*_c_ (W m^−^²K^−1^) is the heat transfer coefficient at the substrate–droplet contact, *T*_b_ (K) is the substrate temperature, *h* (W m^−^²K^−1^) is the heat transfer coefficient at the air–droplet interface, *h*_m_ (m s^−1^) is the mass transfer coefficient at the air–droplet interface, *L*_e_ (J kg^−1^) is the latent heat of evaporation, $\rho_{v,l}$ (kg m^−1^) is the specific mass of saturated water vapour at the droplet surface, $\rho_{v,\infty \;}$ (kg m^−1^) is the specific mass of saturated water vapour at the surrounding air temperature, ε is the droplet surface emissivity, σ (W m^−1^K^−1^) is the Stefan–Boltzmann constant, $T_{\infty }$ (K) is the air temperature, *n*_*r*_ and *n*_*z*_ are the direction cosines of the unit normal vector with the *r* and *z* coordinate axes. Average external heat transfer and mass transfer coefficients were assumed at the air–droplet interface. The typically low values of Biot number (Bi) < 0.1 and the small sizes of the droplet favour the employment of the lumping procedure here proposed, due to the mild temperature variations expected within the droplet. For the same reason, spatial variations inherent to the local heat and mass transfer coefficients are not expected to markedly affect the model’s accuracy. Nevertheless, the correlations for the average coefficients here adopted carry information on the droplet surface temperature, which varies along the droplet height.

Murphy & Kopp [62] provide a comprehensive literature review of correlations for saturated water vapour pressure as a function of temperature. Among the reviewed correlations, the one proposed by Bohren & Albrecht [63] was selected for this study. Consequently, the equations for $\rho_{v,l}\left(T\right)$ and $\;\rho_{v,\infty }$, in terms of the temperature for liquid water are presented below, where RH represents the relative humidity in the air:


(3.2a) $$\rho_{v,l}\left(T\left(r,z,t\right)\right)=\frac{1.323}{T\left(r,z,t\right)}exp\left(19.83-\frac{5417}{T\left(r,z,t\right)}\right)$$

(3.2b) $$\rho_{v,\infty }=RH\left[\frac{1.323}{T_{\infty }}exp\left(19.83-\frac{5417}{T_{\infty }}\right)\right].$$

For the calculation of the convective heat $\left(h\right)$ and mass $\left(h_{m}\right)$ transfer coefficients, correlations for the Nusselt number were taken from Ozisik [64] and for the Sherwood number the equivalent expression was adopted using the Schmidt number instead of Prandtl, as follows.


(3.3a) $$Nu=\frac{2hR_{d}}{k_{\infty }}=2+0.43\;{\left[\frac{g\;\beta_{\infty }D^{3}\left(T_{w}-T_{\infty }\right)}{\nu^{2}}Pr\right]}^{\frac{1}{4}}\;$$

(3.3b) $$Sh=\frac{2h_{m}R_{d}}{D_{a}}=2+0.43\;{\left[\frac{g\;\beta_{\infty }D^{3}\left(T_{w}-T_{\infty }\right)}{\nu^{2}}Sc\right]}^{\frac{1}{4}}\;.$$

The following dimensionless parameters are used:


(3.4) $$\begin{array}{r} \;\theta =\frac{T-T_{\infty }}{T^{*}-T_{\infty }},\theta_{0}=\frac{T_{0}-T_{\infty }}{T^{*}-T_{\infty }},\theta_{b}=\frac{T_{b}-T_{\infty }}{T^{*}-T_{\infty }},\tau =\frac{\alpha t}{L_{2}^{2}},\\ R=\frac{r}{L_{2}},Z=\frac{z}{L_{2}},\gamma =\frac{L_{1}}{L_{2}},R_{w}(z)=\frac{r_{w}(z)}{L_{2}}=\gamma \sqrt{1-Z^{2}} \end{array}.$$

The resulting dimensionless formulation is then written as:


(3.5a) $$\frac{\partial \theta }{\partial \tau }=\frac{1}{R}\frac{\partial }{\partial R}\left(R\frac{\partial \theta }{\partial R}\right)+\frac{\partial^{2}\theta }{\partial Z^{2}}\;at\;[\begin{array}{c} 0<Z<1\\ 0<R<R_{w}(Z)\\ \tau >0 \end{array}.$$

Initial condition


(3.5b) $$\theta \left(R,Z,0\right)=\theta_{0}.$$

Boundary conditions


(3.5c,d) $${\frac{\partial \theta (R,Z,\tau )}{\partial Z}|}_{Z=1}=0;\;\;\;\;{-\frac{\partial \theta (R,Z,\tau )}{\partial Z}|}_{Z=0}+Bi_{c}\theta (0,Z,\tau )=Bi_{c}\theta_{c}$$

(3.5e) $${\frac{\partial \theta (R,Z,\tau )}{\partial R}|}_{R=0}=0$$

(3.5f) $$\left(n_{r}{\frac{\partial \theta (R,Z,\tau )}{\partial R}|}_{R=R_{w}(Z)}+n_{z}{\frac{\partial \theta (R,Z,\tau )}{\partial Z}|}_{R=R_{w}(Z)}\right)+B(\theta )\theta (R,Z,\tau ){|}_{R=R_{w}(Z)}=H_{1},$$

where


$$B\left(\theta \right)=Bi+Bir\left(\beta^{3}\theta^{3}+4\beta^{2}\theta^{2}+6\beta \theta +4\right)+\frac{Bim\rho_{v,l}\left(\theta \right)}{\rho_{v,0}}$$

(3.5g–l) $$\;H_{1}=\frac{Bim\rho_{v,\infty }}{\rho_{v,0}},\;\beta =\frac{T^{*}-T_{\infty }}{T_{\infty }},Bic=\frac{hR_{d}}{k}\;Bim=\frac{h_{m}L_{e}R_{d}\rho_{v,0}}{k_{l}\left(T^{*}-T_{\infty }\right)}\;Bir=\frac{\epsilon \sigma T_{\infty }^{3}R_{d}}{k_{l}}.$$

For the supercooling stage, to simplify the manipulation that follows, the radially averaged dimensionless temperature, $\bar{\theta }\left(Z,\tau \right),$ is written in terms of the auxiliary dependent variable, $\tilde{\theta }\left(Z,\tau \right)$, as:


(3.6a,b) $$\bar{\theta }\left(Z,\tau \right)=\frac{2}{R_{w}^{2}\left(Z\right)}\tilde{\theta }\left(Z,\tau \right)∴\tilde{\theta }\left(Z,\tau \right)=\int_{0}^{R_{w}\left(Z\right)}R\theta \left(R,Z,\tau \right)dR.$$

Thus, the radially averaging operator $\int_{0}^{R_{w}\left(Z\right)}R\_\_\_\_dR$ is applied over equation (3.5a):


(3.7) $$\underset{1st\;term}{\underbrace{\int_{0}^{R_{w}(Z)}R\frac{\partial \theta (R,Z,\tau )}{\partial \tau }\;dR}}=\underset{2nd\;term}{\underbrace{\int_{0}^{R_{w}(Z)}\frac{\partial }{\partial R}\left(R\frac{\partial \theta (R,Z,\tau )}{\partial R}\right)\;dR}}+\underset{3rd\;term}{\underbrace{\int_{0}^{R_{w}(Z)}R\frac{\partial^{2}\theta (R,Z,\tau )}{\partial Z^{2}}\;dR}}.$$

Each term in equation (3.7) is evaluated as follows:

- first term


(3.8a) $$\int_{0}^{R_{w}\left(Z\right)}R\frac{\partial \theta \left(R,Z,\tau \right)}{\partial \tau }dR=\frac{\partial \tilde{\theta }\left(Z,\tau \right)}{\partial \tau }$$

- second term


(3.8b) $$\int_{0}^{R_{w}(Z)}\frac{\partial }{\partial R}\left(R\frac{\partial \theta (R,Z,\tau )}{\partial R}\right)dR=R_{w}(Z){\left.\frac{\partial \theta }{\partial R}\right|}_{R=R_{w}(Z)}$$

- third term


(3.8c) $$\int_{0}^{R_{w}(Z)}R\frac{\partial^{2}\theta }{\partial Z^{2}}\;dR=\frac{\partial^{2}\tilde{\theta }}{\partial Z^{2}}-\left[R_{w}^{\prime }(Z{)}^{2}+R_{w}(Z)R_{w}^{\prime \prime }(Z)\right]\theta {|}_{R=R_{w}(Z)}-2R_{w}(Z)R_{w}^{\prime }(Z)\frac{\partial \theta }{\partial Z}{|}_{R=R_{w}(Z)},$$

where


(3.8d) $$R_{w}^{\prime }{\left(Z\right)}^{2}+R_{w}\left(Z\right)R_{w}^{\prime \prime }\left(Z\right)\equiv d_{1}$$

(3.8e) $$R_{w}\left(Z\right)R_{w}^{\prime }\left(Z\right)\equiv d_{2}.$$

So,


(3.8f) $$\int_{0}^{R_{w}(Z)}R\frac{\partial^{2}\theta (R,Z,\tau )}{\partial Z^{2}}dR=\frac{\partial^{2}\tilde{\theta }}{\partial Z^{2}}+d_{1}\theta {|}_{R=R_{w}(Z)}\;+2d_{2}{\frac{\partial \theta }{\partial Z}|}_{R=R_{w}(Z)}.$$

Replacing equations (3.8a), (3.8b) and (3.8f), into equation (3.7), it results in:


(3.9) $$\underset{\text{1st term}}{\underbrace{\frac{\partial \tilde{\theta }(Z,\tau )}{\partial \tau }}}=\underset{\text{2nd term}}{\underbrace{R_{w}(Z){\frac{\partial \theta }{\partial R}|}_{R=R_{w}(Z)}}}+\underset{\text{3rd term}}{\underbrace{\frac{\partial^{2}\tilde{\theta }}{\partial Z^{2}}+d_{1}{\theta |}_{R=R_{w}(Z)}+2d_{2}{\frac{\partial \theta }{\partial Z}|}_{R=R_{w}(Z)}}}$$

which is reorganized as


(3.10) $$\frac{\partial \tilde{\theta }(Z,\tau )}{\partial \tau }=\frac{\partial^{2}\tilde{\theta }}{\partial Z^{2}}+d_{1}{\theta |}_{R=R_{w}(Z)}+2d_{2}{\frac{\partial \theta }{\partial Z}|}_{R=R_{w}(Z)}+R_{w}(Z){\frac{\partial \theta }{\partial R}|}_{R=R_{w}(Z)}.$$

The last term of equation (3.10) is obtained through the boundary condition, equation (3.5f), which can be rearranged as follows:


(3.11) $${\frac{\partial \theta }{\partial R}|}_{R=R_{w}(Z)}=\frac{H_{1}}{n_{r}}-{\frac{1}{n_{r}}B(\theta )\theta |}_{R=R_{w}(Z)}-{\frac{n_{z}}{n_{r}}\frac{\partial \theta }{\partial Z}|}_{R=R_{w}(Z)}.$$

Applying equation (3.11) into equation (3.10):


(3.12a) $$\begin{array}{rl} & \frac{\partial \tilde{\theta }(Z,\tau )}{\partial \tau }=\frac{\partial^{2}\tilde{\theta }}{\partial Z^{2}}+{d_{1}\theta |}_{R=R_{W}(Z)}+{2d_{2}\frac{\partial \theta }{\partial Z}|}_{R=R_{W}(Z)}+\frac{R_{W}(Z)}{n_{r}}[H_{1}-{B(\theta )\theta |}_{R=R_{W}(Z)}-\\ & {n_{z}\frac{\partial \theta }{\partial Z}|}_{R=R_{W}(Z)}] \end{array}$$

and manipulating the above expression,


(3.12b) $$\begin{array}{r} \frac{\partial \tilde{\theta }(Z,\tau )}{\partial \tau }=\frac{\partial^{2}\tilde{\theta }}{\partial Z^{2}}+\left[d_{1}-\frac{R_{w}(Z)}{n_{r}}B(\theta )\right]{\theta |}_{R=R_{w}(Z)}\\ +\left[2d_{2}-\frac{n_{z}}{n_{r}}R_{w}(Z)\right]{\frac{\partial \theta }{\partial Z}|}_{R=R_{w}(Z)}+\frac{H_{1}}{n_{r}}R_{w}(Z) \end{array}.$$

However, equation (3.12a) has two dependent variables $\theta (R_{w}(Z),Z,\tau )$ and $\tilde{\theta }(Z,\tau )$, besides the derivative of $\theta (R_{w}(Z),Z,\tau )$ in the *Z* variable. Therefore, it is required to relate the external surface temperatures to the radially integrated temperatures, so that one of them can be eliminated from equation (3.12b). To find a relation between $\theta (R_{w}(Z),Z,\tau )$ and $\tilde{\theta }(Z,\tau )$, we will then use Hermite integration formulae to approximate the radially integrated temperatures and heat fluxes, thus employing the so-called CIEA [52,54–58]. Two Hermite approximations shall be here adopted, the H_0,0_∣H_0,0_ and H_1,1_∣H_0,0_ formulae, where H_0,0_ and H_1,1_ correspond, respectively, to the trapezoidal and corrected trapezoidal integration formulae [52,54–58].

For the radially integrated temperature, the Hermite integration formulae, $H_{0,0}$ and $H_{1,1}$, yield:


(3.13a) $$H_{0,0}\to \tilde{\theta }(Z,\tau )=\frac{R_{w}(Z)}{2}\left[{\left.R\theta \right|}_{R=R_{w}(Z)}+{\left.R\theta \right|}_{R=0}\right]$$

(3.13b) $$H_{1,1}\to \tilde{\theta }(Z,\tau )=\frac{R_{w}(Z)}{2}\left[R\theta {|}_{R=R_{w}(Z)}+R\theta {|}_{R=0}\right]+\frac{R_{w}^{2}(Z)}{12}\left({\frac{\partial \theta }{\partial R}|}_{R=0}-{\frac{\partial \theta }{\partial R}|}_{R=R_{w}(Z)}\right).$$

And, for the radially integrated flux, only the $H_{0,0}$ formula will be employed:


(3.13c) $$H_{0,0}\to \theta {|}_{R=R_{w}\left(Z\right)}-\theta {|}_{R=0}=\frac{R_{W}\left(Z\right)}{2}\left(\frac{\partial \theta }{\partial R}{|}_{R=R_{w}\left(Z\right)}+\frac{\partial \theta }{\partial R}{|}_{R=0}\right).$$

The boundary conditions in the R-coordinate are represented by


(3.14a) $${\frac{\partial \theta }{\partial R}|}_{R=R_{w}\left(Z\right)}=\frac{H_{1}}{n_{r}}-\frac{1}{n_{r}}B\left(\theta \right)\theta {|}_{R=R_{w}\left(Z\right)}-\frac{n_{z}}{n_{r}}{\frac{\partial \theta }{\partial Z}|}_{R=R_{w}\left(Z\right)}$$

(3.14b) $${\frac{\partial \theta \left(R,Z,\tau \right)}{\partial R}|}_{R=0}=0.$$

Applying the boundary conditions equations (3.14a) and (3.14b) into equations (3.13a) and (3.13b), one finds:


(3.15a) $$H_{0,0}\to \;\tilde{\theta }\left(Z,\tau \right)=\frac{R_{w}^{2}\left(Z\right)}{2}\theta \left(R_{w}\left(Z\right),Z,\tau \right)$$

(3.15b) $$H_{1,1}\to \tilde{\theta }\left(Z,\tau \right)=\frac{R_{w}^{2}\left(Z\right)}{2}\theta {|}_{R=R_{w}\left(Z\right)}-\frac{R_{w}^{2}\left(Z\right)}{12}\left(\frac{H_{1}}{n_{r}}-\frac{1}{n_{r}}B\left(\theta \right)\theta {|}_{R=R_{w}\left(Z\right)}-\frac{n_{z}}{n_{r}}{\frac{\partial \theta }{\partial Z}|}_{R=R_{w}\left(Z\right)}\right).$$

To find the dimensionless temperature at *R* = 0, we use the boundary condition, equations (3.14a) and (3.14b), into equation (3.13b), to find the expression for the central temperature in terms of the surface temperature:


(3.15c) $$\theta \left(0,Z,\tau \right)=\theta {|}_{R=R_{w}\left(Z\right)}-\frac{R_{W}\left(Z\right)}{2}(\frac{H_{1}}{n_{r}}-\frac{1}{n_{r}}B\left(\theta \right)\theta {|}_{R=R_{w}\left(Z\right)}-\frac{n_{z}}{n_{r}}\frac{\partial \theta }{\partial Z}{|}_{R=R_{w}\left(Z\right)})\;.$$

In this way, one obtains the following lumped-differential formulation using the H_0,0_∣H_0,0_ approximation:


(3.16a) $$\frac{\partial \tilde{\theta }(Z,\tau )}{\partial \tau }=\frac{\partial^{2}\tilde{\theta }}{\partial Z^{2}}+\left[d_{1}-\frac{R_{w}(Z)}{n_{r}}B(\theta )\right]{\theta |}_{R=R_{w}(Z)}+\left[2d_{2}-\frac{n_{z}}{n_{r}}R_{w}(Z)\right]{\frac{\partial \theta }{\partial Z}|}_{R=R_{w}(Z)}+\frac{H_{1}}{n_{r}}R_{w}(Z)$$

(3.16b) $$\tilde{\theta }\left(Z,\tau \right)=\frac{R_{w}^{2}\left(Z\right)}{2}\theta {|}_{R=R_{w}\left(Z\right)}$$

with the following initial condition and boundary conditions:


(3.16c) $$\tilde{\theta }\left(Z,0\right)={\tilde{\theta }}_{0}$$

(3.16d) $${\frac{\partial \tilde{\theta }\left(Z,\tau \right)}{\partial Z}|}_{Z=1}=0$$

(3.16e) $$-{\frac{\partial \tilde{\theta }\left(Z,\tau \right)}{\partial Z}|}_{Z=0}+Bi_{c}\tilde{\theta }\left(0,\tau \right){|}_{Z=0}=Bi_{c}{\tilde{\theta }}_{c}.$$

Similarly, the following lumped-differential formulation is achieved using the H_1,1_∣H_0,0_ approximation:


(3.17a) $$\frac{\partial \tilde{\theta }(Z,\tau )}{\partial \tau }=\frac{\partial^{2}\tilde{\theta }}{\partial Z^{2}}+\left[d_{1}-\frac{R_{w}(Z)}{n_{r}}B(\theta )\right]{\theta |}_{R=R_{w}(Z)}+\left[2d_{2}-\frac{n_{z}}{n_{r}}R_{w}(Z)\right]{\frac{\partial \theta }{\partial Z}|}_{R=R_{w}(Z)}+\frac{H_{1}}{n_{r}}R_{w}(Z)$$

(3.17b) $$\tilde{\theta }(Z,\tau )=\frac{R_{w}^{2}(Z)}{2}{\theta |}_{R=R_{w}(Z)}-\frac{R_{w}^{2}(Z)}{12}\left(\frac{H_{1}}{n_{r}}-\frac{1}{n_{r}}B(\theta ){\theta |}_{R=R_{w}(Z)}-\frac{n_{z}}{n_{r}}{\frac{\partial \theta }{\partial Z}|}_{R=R_{w}(Z)}\right)$$

with the following initial and boundary conditions:


(3.17c) $$\tilde{\theta }\left(Z,0\right)={\tilde{\theta }}_{0}$$

(3.17d) $${\frac{\partial \tilde{\theta }\left(Z,\tau \right)}{\partial Z}|}_{Z=1}=0$$

(3.17e) $$-{\frac{\partial \tilde{\theta }\left(Z,\tau \right)}{\partial Z}|}_{Z=0}+Bi_{c}\tilde{\theta }\left(0,\tau \right)=Bi_{c}{\tilde{\theta }}_{c}.$$

The derivation of the reduced models and the numerical solution of the resulting PDEs as obtained by the CIEA, for both the H_1,1_∣H_0,0_ and H_0,0_∣H_0,0_ approximations, are obtained through a symbolic-numerical code built on the Wolfram Mathematica® v.13.3 platform.

## 4. Results and discussion

### (a) Comparison of experimental and theoretical results

The reduced models, using the H_1,1_∣H_0,0_ and H_0,0_∣H_0,0_ approximations, were symbolically derived and numerically solved with the NDSolve function in the Wolfram Mathematica® v.13.3 platform and compared with the experimental data collected in this study during the supercooling stage of the sessile droplets. To perform this comparison, the input data were obtained from the experiments, complemented by physical data from the literature, as presented in table 2.

**Table 2.** Physical properties and input data.

property	value			source
	SHB	HYB	HYL	
substrate temperature, $T_{c}$ (°c)	−19.7	−18.46	−15.2	experiments
air temperature, $T_{\infty }$ (°c)	−2.8	−2.9	−1.04	experiments
initial droplet temperature, *t*_0_ (°c)	0 ±1	0 ±1	0 ±1	experiments
liquid droplet height, $L_{2}$ (mm)	2.08	1.9	1.55	experiments
liquid droplet base diameter, $L_{1}$ (mm)	2.11	2.87	3.63	experiments
contact angle (°)	130	110	82	experiments
air thermal conductivity, $k_{\infty }$ (W m^−1^ K^−1^)	0.0234			Hindmarsh *et al.* [53]
air diffusivity, *D*_a_	2.060 × 10⁻⁵			Hindmarsh *et al.* [53]
air dynamic viscosity, $\mu_{\infty }$ (Ns m^−2^)	1.663 × 10⁻⁵			Incropera *et al.* [65]
air specific mass, $\rho_{\infty }$ (kg m^−3^)	1.3317			Hindmarsh *et al.* [53]
volumetric expansion coefficient, $\beta_{\infty }$(1 K^−1^)	0.034			Incropera *et al.* [65]
liquid specific heat, $c_{l}$ (kJ kg^−1^ K^−1^)	4.2			Hindmarsh *et al.* [53]
liquid thermal conductivity, $k_{l}$ (W m^−1^ K^−1^)	0.607			Incropera *et al.* [65]
latent heat of evaporation, $L_{e}$ (J kg^−1^)	2.502 × 10⁶			Hindmarsh *et al.* [53]
emissivity, ε	0.96			Hindmarsh *et al.* [53]
liquid specific mass, $\rho_{l}$(kg m^−3^)	1000			Hindmarsh *et al.* [53]
specific mass of sat. water vapour, $\rho_{v,o}$ (kg m^−3^)	4.8473 × 10⁻³			Hindmarsh *et al.* [53]
specific mass of sat. water vapour at the surface, $\rho_{v,l,s}$ (kg m^−3^)	equation (3.2b)			Hindmarsh *et al.* [53]
Stefan–Boltzmann constant, σ(W m^−2^ K^−4^)	5.670 × 10⁻⁸			Incropera et al. [65]

**Figure 4.**
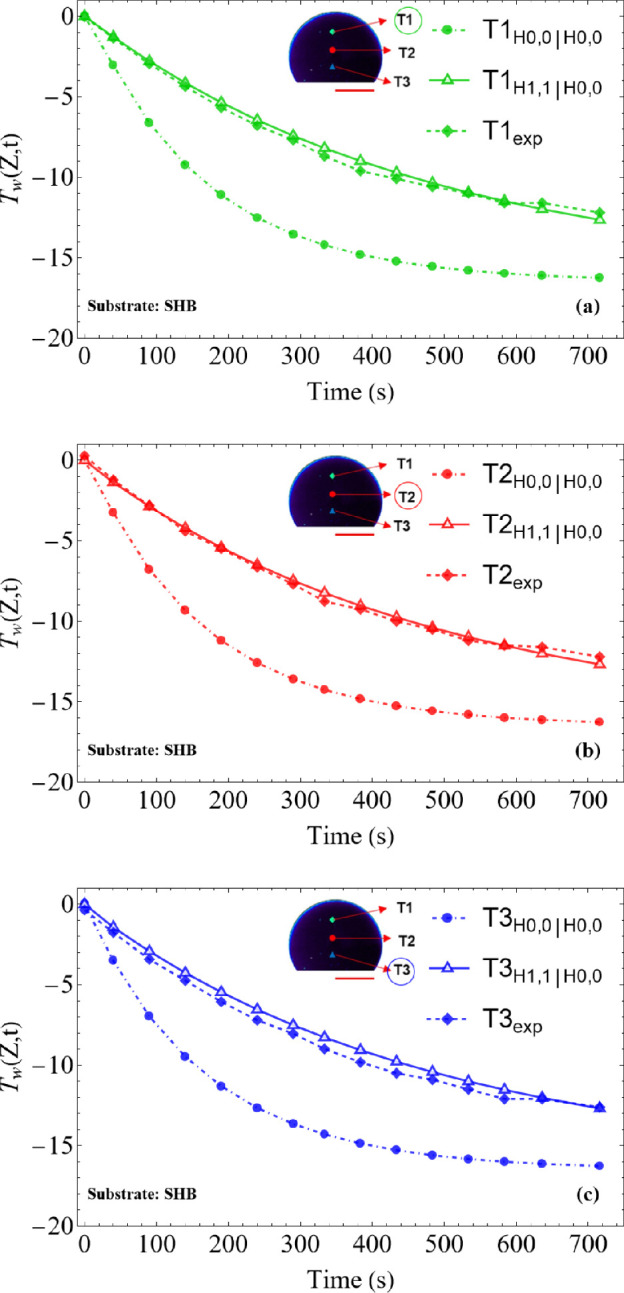
Temperature variation at the sessile droplet surface on SHB substrate for cooling stage: comparison between theoretical (CIEA: H_0,0_∣H_0,0_ and H_1,1_∣H_0,0_) and experimental results: (a) T1 (at 3/4 *h*_0_), (b) T2 (at 1/2 *h*_0_), and (c) T3 (at 1/4 *h*_0_). $L_{1}=2.11\;\text{mm},\;L_{2}=2.08\;\text{mm},\;\;T_{b}=-19.7,\;T_{\infty }=-2.8{\;}^{\circ }\text{C}$ and *T*_w_(*Z*,*t*) at the droplet surface.

**Figure 5.**
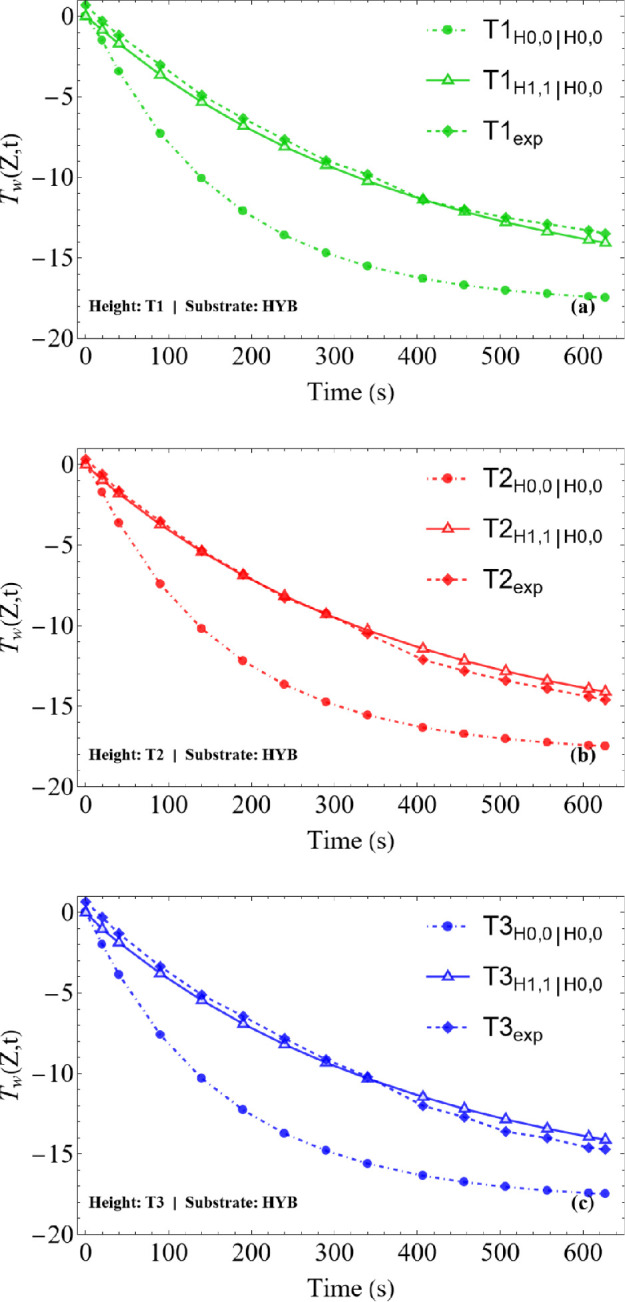
Temperature variation at the sessile droplet surface on HYB substrate for cooling stage: comparison between theoretical (CIEA: H_0,0_∣H_0,0_ and H_1,1_∣H_0,0_) and experimental results: (a) T1 (at 3/4 *h*_0_), (b) T2 (at 1/2 *h*_0_), and (c) T3 (at 1/4 *h*_0_). . $L_{1}=2.87\;\text{mm},\;L_{2}=1.9\;\text{mm},\;\;T_{b}=-18.46^{\circ }\text{C},\;T_{\infty }=-2.9{\;}^{\circ }\text{C}$ and *T*_w_(*Z*,*t*) at the droplet surface.

**Figure 6.**
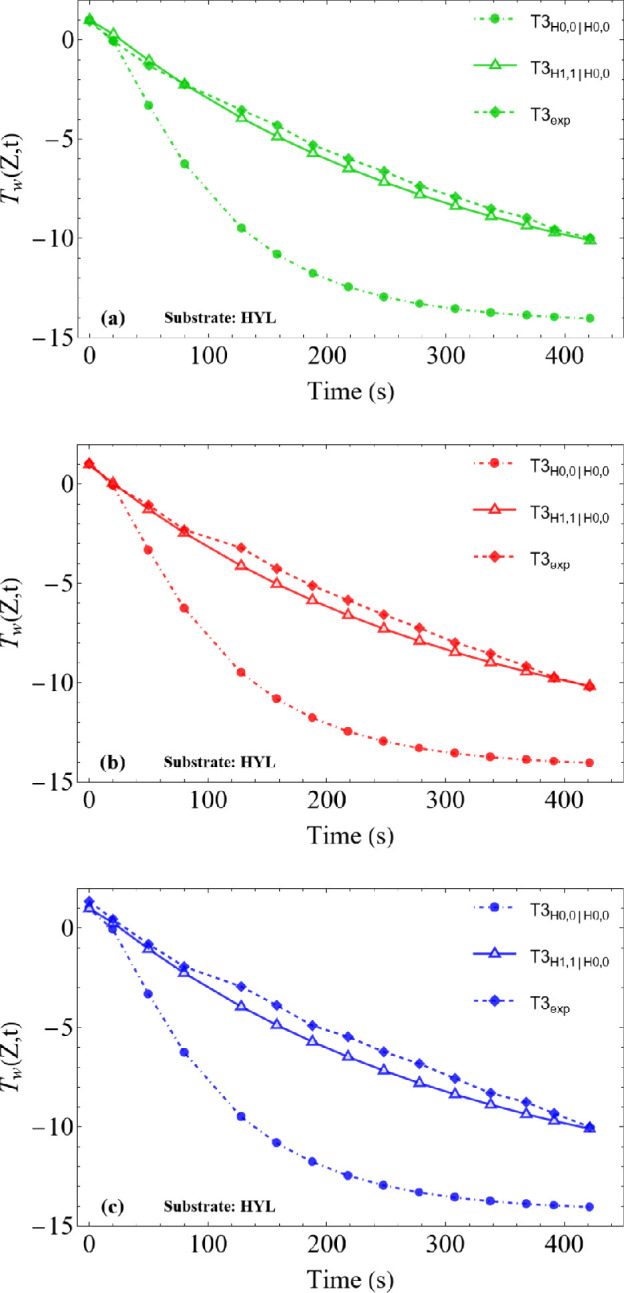
Temperature variation at the sessile droplet surface on HYL substrate for cooling stage: comparison between theoretical (CIEA: H_0,0_∣H_0,0_ and H_1,1_∣H_0,0_) and experimental results: (a) T1 (at 3/4 *h*_0_), (b) T2 (at 1/2 *h*_0_), and (c) T3 (at 1/4 *h*_0_). $L_{1}=3.63\;\text{mm},\;L_{2}=1.55\;\text{mm},\;\;T_{b}=-15.2^{\circ }\text{C},\;T_{\infty }=-1.04{\;}^{\circ }\text{C}$ and *T*_w_(*Z*,*t*) at the droplet surface.

The analysis was conducted for the three different substrates (HYB, SHB and HYL), detailed in §2 and at three distinct positions along the droplet height: T1 (located at 3/4 of the initial droplet height, *h*_0_), T2 (at 1/2 *h*_0_) and T3 (at 1/4 *h*_0_), as shown in figures 4–6.

It is observed that the results obtained using the H_0,0_∣H_0,0_ approximation, which essentially reduces to the classical lumped system analysis, show a significant deviation from the experimental data. This occurs because this approximation assumes that the droplet temperature along the radial coordinate is practically uniform, or that the surface temperature is essentially equal to the radial average, which appears to be a poor approximation in this problem. On the other hand, the H_1,1_∣H_0,0_, approximation, which incorporates more information from the boundary condition at the droplet surface (see equation (3.15b)), thus rewriting the surface temperature as a function of the radially averaged temperature, shows excellent agreement with the experimental results. The differences observed between the simulated and measured values remain within the 1°C uncertainty of the measurements (infrared camera), validating the theoretical model in predicting the thermal behaviour of the droplet, regardless of the wettability of the substrate (hydrophilic HYL, hydrophobic HYB and superhydrophobic SHB substrates).

Additionally, figures 4–6 show that the droplets start the supercooling stage at an initial temperature close to 0°C and, over time, cool down until they reach the nucleation temperature. This point marks the onset of the recalescence stage, when latent heat is released and then freezing starts, temporarily halting the temperature drop. The nucleation times, which vary depending on the substrate, were used as a stopping criterion in the simulations: 716 s for the SHB substrate, 626 s for HYB and 422 s for HYL. The above comparisons provide a co-validation of the experimental and theoretical results and encourage pursuing further analysis towards the simulation of the freezing and final cooling stages.

### (b) Temperature variation during nucleation and freezing of the sessile droplet

**Figure 7.**
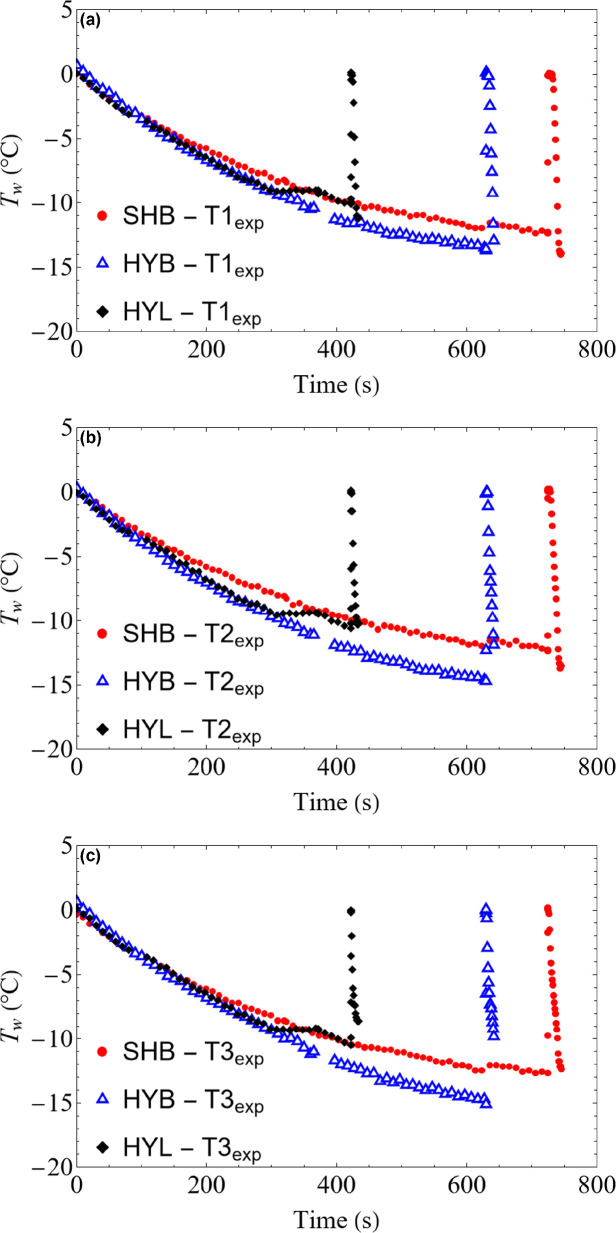
Evolution of the droplet surface temperature, *T*w, on three different substrates where SHB is superhydrophobic (plot marker circle), HYB is hydrophobic (plot marker triangle) and HYL is hydrophilic (plot marker diamond). Three locations are chosen for each of the substrates. They are (*a*) top plane (T1_exp_), (*b*) middle plane (T2_exp_) and (*c*) bottom plane (T3_exp_). The subscript ‘exp’ is for the experimental origin of the plotted data.

Using the IR thermography videos, the surface temperature history (*T*_w_(*Z*,*t*)) at three different planes (T1_exp_, T2_exp_ and T3_exp_) of the droplet is plotted for different substrate wettability, based on a single experiment. Repeating this experiment is highly challenging due to difficulties in controlling the initial droplet and ambient conditions figure 7, in addition to the probabilistic nature of the freezing onset itself. Therefore, the different experimental runs cannot be considered actual replicas of the experiment. Nonetheless, three experimental runs were conducted, and deviations in the temperature readings in individual runs with respect to the average value during the cooling stage were observed, varying between 0.2 and 0.8°C, depending on the droplet shape. Observed variation in *T*_w_ for the three substrates (figure 7*a*–*c*) is expected due to dependence of heat transfer on the droplet geometry and the relative distance of the temperature plane from the substrate. This variation is accurately modelled, for the supercooling stage of the droplet as shown in the preceding section (see figures 4–6). Additional factors also play a role, such as variations in radiative transfer view factor [66] due to the droplet’s curvature (see §2), being different for hydrophobic and hydrophilic droplets. Variations in the ambient temperature also contribute to the temperature-dependent convection and radiation exchange with the environment. These factors eventually affect the onset of the recalescence stage (figure 7) when the droplet’s temperature rises sharply to 0.6–0.8°C, higher than the expected equilibrium temperature of 0°C. Clearly, the onset of freezing is significantly earlier for HYL than either HYB or SHB due to the reduced energy barrier of nucleation and is consistent with the literature [36].

**Figure 8.**
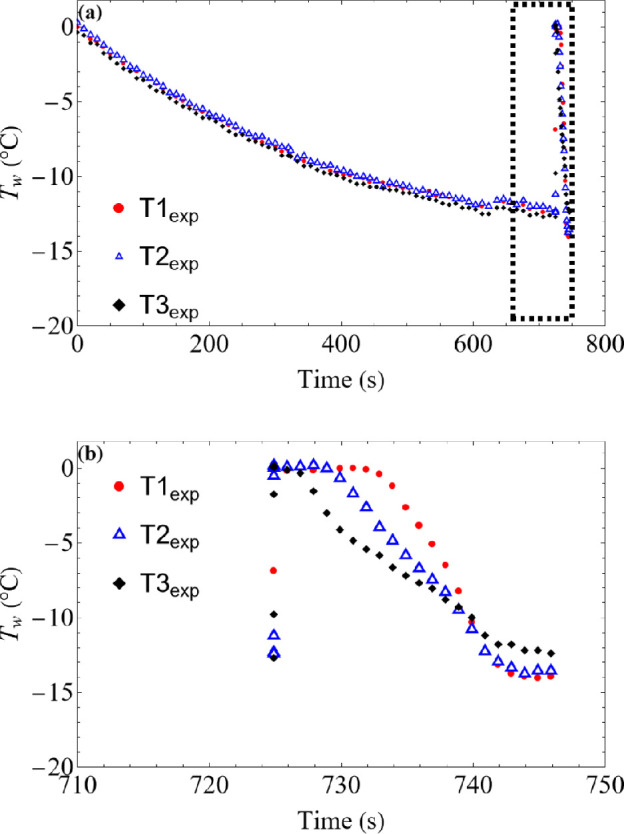
Surface temperature variation for the sessile droplet on SHB substrate. (*a*) Temperature variation at three different locations (T1, T2, T3) for the entire duration of cooling and freezing stages (recalescence is indicated in the dashed bo); (*b*) dashed box is magnified and replotted.

Next, the dynamics of recalescence and solidification are better elucidated by measuring the temperature variation at different heights of the same droplet (SHB), as shown in figure 8*a*. The rise in droplet temperature is first observed in T3_exp_ (bottom most location). This is expected due to the higher probability of heterogeneous nucleation triggering at the droplet–substrate interface [29]. The temperature rise for T2_exp_ and T1_exp_ are 5 and 10 ms later (not visible on the plot), respectively. Given the known distance between T1_exp_, T2_exp_ and T3_exp_, a rough estimate of the average recalescence speed is found to be *O* (10^2^–10^1^) mm s^−1^, slightly higher than previously reported values [25,34]. The overestimation is possibly due to the limited temporal resolution of the IR imaging.

In the second stage of freezing, the temperature at any given location in the droplet remains constant till the freezing front moves past this location, as shown in figure 8*b*. In case of the SHB surface shown above, the onset of temperature fall at T1_exp_, T2_exp_ and T3_exp_ is nearly 3, 7 and 11 s post-recalescence, respectively. The average speed of the icing front is approximately *O* (10^−1^) mm s^−1^. However, the solidification rate is not constant throughout the stage as discussed in the next section.

### (c) Dynamics of ice growth

**Figure 9.**
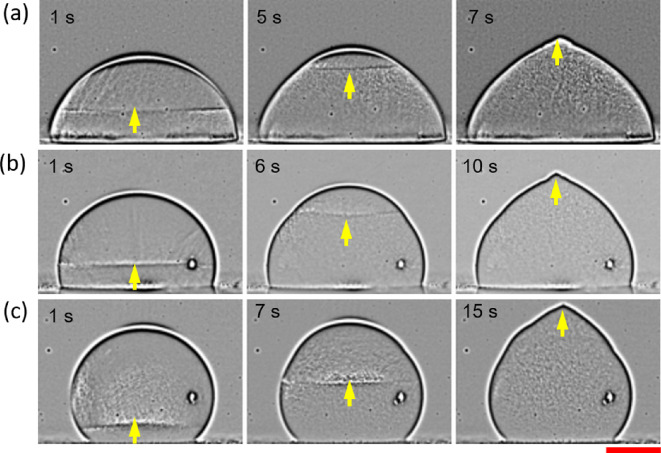
Snapshots showing solidification for substrates with different wettability: (*a*) HYL (1, 5, and 7 s), (*b*) HYB (1, 6, and 10 s), and (*c*) SHB (1, 7 and 15 s). The arrow indicates the position and direction of ice front development. Time stamp is inset in the top-left corner. Scale bar equals 1 mm.

High-speed optical images are used to track the ice front (see §2). The images are spatially band-pass filtered using the FFT plugin in ImageJ (open source image processing software) to remove regions of extreme contrast and improve clarity. Figure 9*a*–*c* show the growing ice front at different time instants. The final morphology of the droplet is characterized by a pointy tip at the apex position, irrespective of the substrate wettability. This is caused by expansion of water on freezing and the curving of the ice front near the droplet’s edge, not visible here due to limitations of the imaging system. Marin *et al.* [32,33] used a Hele-Shaw arrangement to visually confirm the front’s curving and argued that the front itself is a part of a larger circle centred at the apex. As the front progresses, the radius of the front (ice–liquid contact) reduces and finally vanishes into a singular tip at the apex.

**Figure 10.**
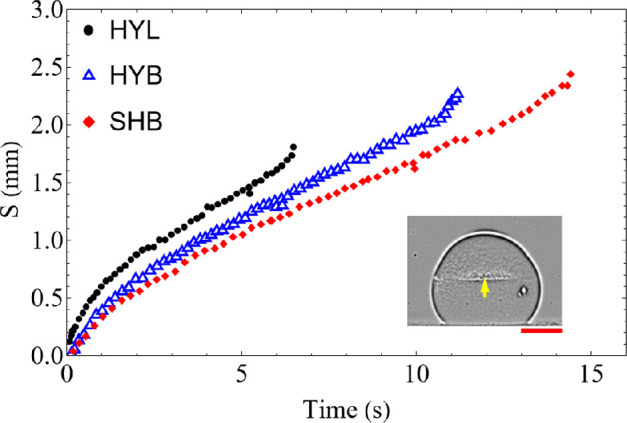
Position of the ice front (*S*) relative to the substrate as a function of time for experiments on three different substrates.

The freezing front development for the three substrates is plotted in figure 10, for a single experiment, since it is extremely difficult to perform repetitions of this experiment due to the challenges in controlling the shape of the droplet and initial and control conditions, as previously mentioned (§4b). However, three experimental runs were conducted, and the front position deviations of the individual runs vary from 0.02 to 0.2 mm, calculated relative to the mean value of the experiments conducted. Since the temperature difference between the freezing front and solid substrate remains roughly constant as the height of solid mass increases, the temperature gradient decreases due to the increase in thermal resistance offered by the thicker solid layer, which is however counterbalanced by the decreasing solid–liquid contact area after the largest radius cross section. Since the solidification rate is governed mainly by heat dissipation to the substrate, the front speed slows down in the first stages, as observed in figure 10, followed by a more or less constant value in the intermediate stage, and then markedly accelerating towards the final stages, due to the vanishing water–ice interface [24] and thus vanishing liquid water volume. The hydrophilic droplets have a slightly higher rate of ice progression compared with the hydrophobic cases. This is consistent with the numerical model developed by Zhang *et al.* [31].

## 5. Conclusions

Supercooling and freezing of sessile droplets deposited over colder substrates have been studied both experimentally and theoretically. Non-intrusive temperature measurements at the droplet surface by IR thermography, together with high-speed camera images, have provided valuable data for the analysis of the heat transfer process and the detailed dynamics of the recalescence phenomena, of the solidification front movement, and of the droplet shape changes. The study also explored the three distinct ranges of substrate wettability, namely hydrophilic, hydrophobic and superhydrophobic, to investigate the differences in the phase change process. The onset of freezing was observed earlier on the hydrophilic substrate due to lower energy barrier to nucleation. The speed of recalescence is *O* (10^2^–10^1^) mm s^−1^ while the speed of the solidification is *O* (10^−1^) mm s^−1^. The analysis also provided a co-validation of measurements and simulations from a proposed reduced model for the supercooling stage. A problem reformulation strategy was employed known as the CIEA, which is essentially an improved lumping procedure $\left({\text{H}}_{\text{1,1}}|{\text{H}}_{\text{0,0}}\;approximation\right)$ that approximates averaged temperatures and heat fluxes using Hermite formulae for integrals, instead of just the classical lumped system assumption of uniform radial temperature distributions$\left({\text{H}}_{\text{0,0}}|{\text{H}}_{\text{0,0}}\;approximation\;in\;the\;present\;case\right)$. The latter model leads to significant error since the assumption of a uniform radial temperature is physically inadequate. The present work shall form the basis for the proposition of more complete models that account for the recalescence and freezing stages, as well as for the droplet deformation along the process. Experiments should now proceed to different liquids, suspensions and droplet sizes and shapes, as well as to further understanding the interactions with the treated substrate.

## Data Availability

The data are available from Figshare at [67].
